# 

**Published:** 1825-02

**Authors:** 


### MONTHLY CATALOGUE OF MEDICAL BOOKS.

*** It having been hinted to us that gentlemen, sending copies of their Works, prefer having their titles given at length, it is our intention, in future, to comply with this suggestion: but it is to be observed, that no books can be entered on this List except those sent to us for the purpose;—finding, in the lists hitherto transmitted, that the names of works have frequently been given as published, which have not appeared for weeks, or even months after.*

Analysis of Medical Evidence; comprising Directions for Practitioners in view of becoming Witnesses in Courts of Justice, and an Appendix of Professional Testimony. By JOHN GORDON SMITH, M.D.—London, 1825; pp. 386.

The Anatomy of the Brain, adapted for the use of Students; comprising Directions with regard to the Method to be pursued in its Dissection, conformable to the Mode practised by most Anatomists.—London, 1824; pp. 162.

A Compendium of Theoretical and Practical Medicine; comprising, with the Symptoms, Diagnosis, Prognosis, and Treatment of Diseases, a general Review of Physiology and Pathology; together with an Estimate of the present State of Medical Science. By DAVID UWINS, M.D. Licentiate of the Royal College of Physicians, London; late President of the London Medical Society; Corresponding Member of the Medico-Chirurgical Society of Berlin; Member of the Society of Physicians of the United Kingdom; Lecturer on the Theory and Practice of Medicine; and Physician to the City Dispensary.—London, 1825; pp. 386.

Opinions on the Causes and Effects of the Disease denominated Tic Douloureux; deduced from practical Observations of its supposed Origin, in Lateral Pressure, Distortion, or undue Contact in the Teeth; but more particularly those nearest the Maxillary Sinus, and thence conveying its distressing Sensations to the more distant Extremities of the System. The whole illustrated by three Lithographic Engravings, with annexed Cases confirmatory of the Opinions and Suppositions; as also a peculiar and easy Mode of Ascertainment and Cure. By CHARLES BEW, Surgeon-Dentist to his Majesty, and Royal Household, and also to their Royal Highnesses the Duke and Duchess of Clarence.—London, 1824; pp. 94.

---

### NOTICE TO CORRESPONDENTS.

*We have received the Communications of* Mr. POLLOCK, Dr. BELCHER, Dr. NICHOLLS, Dr. KINGLAKE, Mr. ANDERSON, *and* Dr. CORBAN.

*The envelope in which the Paper on Gastrotomy was inclosed, has been mislaid: we shall feel much obliged to the author if he will favour us with his name as soon as convenient.*

*The case of Hysteria, dated July 8th,* 1824, *is very well marked and well detailed, but it is of every-day occurrence, and therefore unfit for publication.*

*We are obliged to* Mr. MILES *for his Letter: it came to hand too late for us to avail ourselves of it.*

*Our Correspondent at Preston will perceive that we have attended to his request.*

